# Retrospective study of the incidence of unstable and shock patients presenting to the emergency room

**DOI:** 10.1186/2045-4015-3-34

**Published:** 2014-10-28

**Authors:** Sody A Naimer, Moshe Y Prero, Tamar Freud, Carmi Bartal

**Affiliations:** Elon Moreh Health Center, Clalit Health Services, Shomron, Israel; Siaal Research Center for Family Medicine and Primary Care, Faculty of Health Sciences, Ben-Gurion University of the Negev, Beer-Sheva, Israel; Emergency Medicine Department, Soroka Medical Center, Ben-Gurion University of the Negev, Beer-Sheva, Israel; The Medical School for International Health, Faculty of Health Sciences, Ben-Gurion University of the Negev, Beer-Sheva, Israel; Department of Family Medicine, Faculty of Health Sciences, Ben-Gurion University of the Negev, POB 653, Beer-Sheva, 84105 Israel

**Keywords:** Unstable patients, Altering rates, Emergency room, Intensive care, Shock room

## Abstract

**Background:**

Over a period of three decades, medical personnel working in our emergency room observed that fewer severe cases presented to the emergency department. The objective of this study is to assess whether a genuine change in the presentation rates of clinically unstable non-trauma patients to the emergency room indeed exists.

**Methods:**

We conducted a retrospective review of patients treated in the shock room. Patients’ demographic data, diagnoses and outcomes were accessed. Populations of patients presenting to the shock room over a span of four seasons, in two separate periods eight years apart were compared. This rate was compared with the complementary bulk rate of patients presenting to the emergency room at the center.

**Results:**

While absolute rates of emergency room utilization rose, the rate of unstable patients demanding urgent intensive care showed a clear decline. An absolute reduction of close to 50% across the different seasons of the examined years was found. Per patient, the proportion of those requiring artificial respiration and urgent hemodialysis remained uniform in both periods. All parameters of patient outcomes were similar in both periods of the study.

**Conclusion:**

This unexplored aspect of emergency care demonstrates a dramatic decline in the incidence of unstable patients. While we should continue to reinforce delivery of superior care, our medical educational system should adapt itself to compensate for the diminished exposure of our trainees to emergencies. Further research in this field should explore whether the trend we observed exists in other geographical locations and whether this parameter can be utilized as a quality measure of medical systems.

## Background

Medical personnel working in the emergency room for the past thirty years can testify to dramatic shifting trends in the frequency of severe cases presenting to the emergency department. Medical conditions frequently encountered in the past, such as status asthmaticus and status epilepticus, are presently rarely encountered [1, 2]. Ketoacidotic patients and those with decompensated cardiac states appear less frequently. Conversely, the frequency of patients with systemic inflammatory response syndromes is on the rise [3, 4]. However, the emergency room workload exhibits a steady and substantial increase. This fact may be readily explained by the recent rise of longevity, carrying with it the consequences of chronic morbidity [5]. We were under the impression that we were seeing fewer critical patients arriving at our institutions and this trend seems to be continuing even now. Tedious efforts failed to reveal previous literature pertaining to altering trends in the frequency of such cases. Therefore we decided to perform a scientific appraisal of whether changes in the rate of presentation of unstable patients at our center are authentic.

## Methods

### Design

We conducted a retrospective review of all patients treated in the emergency room who were diagnosed with defined unstable conditions unrelated to trauma presenting at the major medical center (Soroka University Medical Center) of southern Israel. The studied shock room constitutes the single destination for all unstable subjects in a peripheral region with a population of over one million people. This hospital is also the only level 1 trauma center in the region and serves as the training center of medical students from the medical faculty of the Ben Gurion University of the Negev. The entire population receiving care at the medical center holds equal, satisfactory health insurance as mandated by the law of the country.

The study was approved by the institutional review board of the Soroka Medical Center.

### How the study group was selected

Patients were exclusively all those admitted to the shock room during the chosen study periods. Intensive care flow sheet recording basic hemodynamic data as blood pressure, pulse and oxygen saturation and the treatment administered are archived in designated files. These were accessible as we embarked on this study. The year 1999 was chosen for earliest reference since documentation before this year is unavailable. The selected months were chosen to reflect the burden of fluctuating morbidity during the different seasons of the year. Demographic data, major diagnosis during hospitalization, and outcome was accessed through a computerized database. Complete original hand written patient files were not accessible through the computer system. We would like to stress that the criteria for treating patients in the shock room are rigid. The Australasian triage score (ATS), in use since 1994, [6] has been applied in our emergency room without change since 1997. Triage is performed by trained nurses upon entrance to the emergency room. The staff during this 8 year period remained uniform. Senior physicians remained in their positions with younger interns using regulation admission policies. Category 1 patients include all states of unstable vital signs or urgent need for full attention of the medical team to prevent complications, late sequelae or death. These cases are sent directly to the shock room. Examples of category 1 diagnoses include:

Patients with severe respiratory compromise on the verge of endotracheal intubation or arrival after prehospital artificial respiration.

Hemodynamic collapse of all etiologies: sepsis, severe allergic reactions, extreme tachy/brady-arrhythmias.

Unconscious states of diverse etiology such as: intracerebral hemorrhage, acidosis, hypothermia etc.

Acute intoxication with metabolic aberration (our center treats tourists who are victims of the unfortunate events of Dead Sea water ingestion [7, 8].

### Main outcomes measured

We utilized the hospital tracking system (a computerized system connected to the primary care service in the community) to collect demographic data, major diagnoses, survival, and general outcomes. The data was compared with complementary presentation rates to the general emergency department. Four different months of the year were compared in an attempt to compensate for possible fluctuations of disease prevalence owing to seasonal variability. Groups of diseases were cumulated into etiological clusters of conditions. Procedures patients underwent were charted. Information retrieval was performed by a computer technician uninformed about the objectives of this research. Diagnoses and medical interventions were routinely fed into the computer bank long before embarking on this trial. Treatment duration was measured from time of arrival at the shock room until transfer to the hospital ward. Population size statistics were sourced from the Central Bureau of Statistics.

### Data analysis

Sociodemographic variables such as gender and race are displayed as frequency and percentages (Table 1). Continuous variables such as age are displayed as mean ± standard deviation (SD). Differences in patients’ characteristics were compared for significance between study periods by using chi-square or Fischer’s exact test as appropriate for categorical variables and by univariate analyses and one-way anova for continuous variables. P values less than or equal to 0.05 were considered statistically significant. All analyses were conducted using SPSSTM 17.0 (Statistical Package for the Social Sciences).

**Table 1:** **Comparison of socio-demographic characteristics of patients visiting emergency department shock room**

	Period				P value
	I		II		
	Nov 1999-Jul 2000 (N = 277)		Nov 2007-Jul 2008 (N = 140)		
	N	%	N	%	
**Gender**					
Male	146	52.7%	80	57.1%	
Female	131	47.3%	60	42.9%	0.225
	277		140		
**Age**					
Mean ± Std	69.5 ± 17.1		63.8 ± 20.4		
Range	0-95		3-97		0.003
	277		140		
**Living place**					
Bedouin encampments	18	6.6%	22	16.3%	0.004
City	235	85.8%	98	72.6%	
Other	21	7.7%	15	11.1%	
	274	(mis = 3)	135	(mis = 5)	
**Nationality**					
Jews	226	92.2%	98	79.0%	
Other	19	7.8%	26	21.0%	0.0004
	245	(mis = 32)	124	(mis = 16)	

## Results

**Figure 1:**
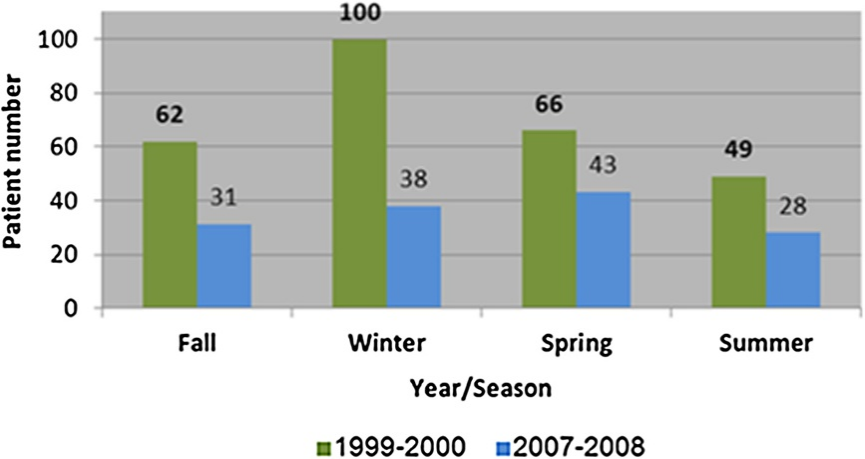
**Comparison of number of patients admission to the emergency department shock room by year and season.**

**Figure 2:**
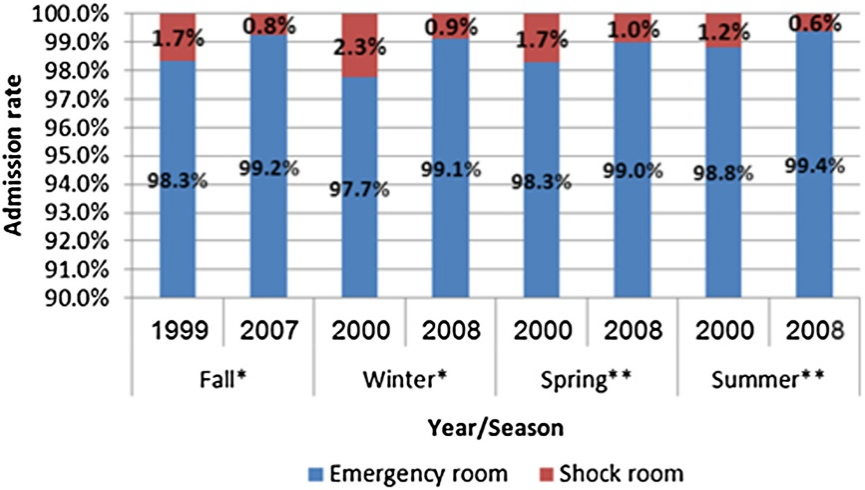
**Comparison of admissions to the emergency department (shock room/emergency room) by year and season.** *p < 0.0001, **p < 0.01.

In this study we examined the number of admissions to the shock room in the emergency department at the Soroka Medical Center during the four seasons of years 1999 - 2000 (period 1) and 2007-2008 (period 2). Despite the delay of this report due to technicalities, our medical staff continues to observe the reported trend albeit with recent relative stabilization. We observed that the number of medically unstable subjects presenting to the emergency department decreased by close to 50% (276 in ′99-′00 and 139 in ′07-′08). Figure 1 shows the comparison of the number of patients admitted to the emergency department shock room by year and season. In each season a statistically significant difference was found between the rates of emergency department shock room arrivals. Figure 2 shows the comparison of shock room admission rates to those of patient referrals to the general emergency department in each year and season. The proportion of shock room treated patients was significantly lower in period 2.

### Population characteristics

The population of the Negev (Southern Israel region receiving care at our medical center) was 872,200 in period 1 during which there were 16145 referrals to the non-trauma emergency department. In period 2 the total population reached 1,067,400, during which there were 17,134 visits. There was no change in gender rates between the periods, though statistically significant differences were observed in patients’ age, living place and nationality between the two study periods (Table 1). The subjects in the earlier period were older and comprised of less Bedouins from the rural areas. The mean age of the Bedouin tribe unstable patients was uniformly 52 years old, consistently younger than the unstable Jewish subjects with a mean age of 71 years old and 67 years old respectively. During this period the mean age of the Negev population increased from 56 to 58 years and the percentage of the elder population (above 75 years) increased from 12% to 15% [9].

### Pathogenesis of unstable patients

Significant statistical differences between periods were also found in the prevalence of major diagnoses: a decrease was found in the incidence of cardiac diagnoses (37.9% vs. 22.3%, p = 0.002) and an increase was seen in the prevalence of toxic and metabolic diagnoses (9.1% vs. 19.0%, p = 0.006). The incidence of respiratory and central nervous system diagnoses or infectious disease causes for instability remained uniform. Major interventions performed in each group demonstrated similar rates of artificial respiration (one third) and urgent hemodialysis (as low as 1-2%) in groups of both periods (Table 2).

**Table 2:** **Comparison of major procedures in unstable patients i.e. artificial respiration and hemodialysis in both groups**

	Period				P value
	I		II		
	Nov 1999-Jul 2000 (N = 277)		Nov 2007-Jul 2008 (N = 140)		
	N	%	N	%	
**Artificial respiration**					
Yes	75	27.1%	47	33.6%	0.104
No	202	72.9%	93	66.4%	
	277		140		
**Hemodialysis**					
Yes	1	0.4%	1	0.7%	0.559
No	276	99.6%	139	99.3%	
	277		140		

### Shock room visit outcome

Comparison of outcomes of patients treated in the emergency department shock room between both study periods are shown in Table 3. Therapeutic detention was on average longer in period 2 compared to period 1 (1.34 ± 1.29 vs. 1.65 ± 1.56, p = 0.025). The difference in patient death rate in the emergency room (6.1% vs. 10.7%, p = 0.097) was of no statistical significance. Similarly, the overall survival and discharge rates remained stable in both periods.

**Table 3:** **Comparison of visit characteristics and outcomes of patients visiting the emergency department shock room**

	Period				P value
	I		II		
	Nov 1999-Jul 2000 (N = 277)		Nov 2007-Jul 2008 (N = 140)		
	N	%	N	%	
**Treatment duration (hours)**					
Mean ± Std	1.34 ± 1.29		1.66 ± 1.56		0.025
Range	0-7.7		0-9.8		
	277		140		
**Major diagnosis**					
Cardiac	96	37.9%	27	22.3%	0.002
Respiratory	48	19.0%	20	16.5%	0.566
Infectious disease	52	20.6%	30	24.8%	0.353
Central nervous system	24	9.5%	9	7.4%	0.513
Toxic metabolic	23	9.1%	23	19.0%	0.006
Dead on arrival	10	4.0%	12	9.9%	0.02
	253	(mis = 27)	121	(mis = 20)	
**Shock room release status**					
Hospitalized/Discharged home from hospital the day of arrival	260	93.8%	125	89.2%	0.097
Died in emergency room	17	6.1%	15	10.7%	
	277		140		
**Hospitalization duration (days)**					
***Transferred to other institutions***					
Mean ± Std	24.75 ± 19.14		15.0±		0.680
Range	12-53		15-15		
	4		1		
***Died during hospitalization***					
Mean ± Std	8.5 ± 11.34		6.43 ± 9.22		0.281
Range	1-67		1-55		
	82		49		
***Discharged***					
Mean ± Std	9.68 ± 16.29		12.40 ± 15.38		0.229
Range	1-145		1-109		
	168		72		

## Discussion

While many aspects of emergency medicine have been extensively explored, the actual burden and magnitude of the critically ill presenting for urgent medical care has been examined far less closely. At a glance, this parameter may reflect the efficacy of both community and health center care, as well as emergency pre-hospital services.

We find the demonstrated decrease in genuine rates of unstable patients highly unexpected given the fact that our emergency department serves the entire Negev region whose population has increased by 22.4% in the course of these 8 years of the study. This increase includes all age groups [10, 11]. The relative component of the Bedouin sub-population rose from 23% at the beginning, to 28%. The advantage of the constellation of this study is that the chosen shock room constitutes the single destination for all unstable subjects arriving from both rural and urban periphery of a population of over one million people. Thus confounding by arbitrary patient alteration of selection of different medical centers over a course of time cannot exist. All unstable subjects that do not succumb to their condition before evacuation will find themselves in the same beds of the shock room in each of the compared years of the study.

We witnessed a general rise of quality care health measures in parallel to the elevation of life expectancy in this period. Since those included in the shock room treated group were not necessarily in the much older age bracket, we readily accept that the final common pathway to instability is steadily on a downward slope and generally, better health is dominant in our communities. How can we explain the disparity between population growth, aging and the decrease in admissions to the shock room?

Three studies of European populations, performed in the course of this study support the optimistic view that dementia risk may be decreasing among older adults [12–14]. The authors hypothesize that these changes were attributable to secular changes in education, population level reductions in vascular risk factors, and an overall reduction in stroke incidence.

In a survey of 26 medical centers across Israel, the Acute Coronary Syndrome Israeli Survey showed a decrease in the number of patients hospitalized due to acute coronary syndrome from 1,794 in 2000 to 1,746 in 2008 [15].

The availability of newer technologies and medications for chronic diseases probably contributes to the stabilization of chronic disease. Billings et al demonstrated that hospital admission rates for conditions which are sensitive to ambulatory care are indicative of high quality preventative and outpatient care for chronic diseases [16, 17]. Unfortunately they did not examine the component of unstable patient admission separately. Primary care has undergone major changes particularly in the Negev. During the 8 year period the number of specialists in primary care increased from 370 to 480 (a 27% increase) and 18 new family health centers were established, 80% of them in the Bedouin sector. Overall emergency room utilization rate dropped from a ratio of 1.85% in period 1 to 1.61% in period 2. Additional evidence supporting better community care indicates that in the past decade, primary presentation of diabetic patients as an event of diabetic ketoacidosis has dropped in the Negev from 30% to a remarkable low level of 17%. Barski et al summarizing a series of ketoacidosis events, claim that there is a steady decline in the proportion of primary presentations of ketoacidosis to our institution [18]. Accessibility to primary care is a factor that has been shown to reduce exacerbations and deterioration to an unstable status [19].

Within the period of this study new pre-hospital emergency vehicle dispatch centers were opened. As opposed to cases of unstable trauma cases where better trained personnel provide little or no advantage [20], teams including well trained paramedics, applying prepared codes and fully equipped with diagnostic and therapeutic means to deliver full advanced lifesaving, care can make a major impact on outcome by stabilizing patients before they reach the medical center [21]. The health status of members of traditional tribes living in rural areas tends to be poorly controlled, and their health is more likely to deteriorate -- either because of limited access or lack of knowledge, diminished faith in the healthcare system, or limited resources [22]. Furthermore, fully qualified personnel that can competently diagnose and treat myocardial infarctions or life threatening arrhythmias can now apply a fast track admission directly to the intensive cardiac unit. New stations particularly in the vicinity of Bedouin encampments may explain their greater representation as unstable patients.

An explanation for the rise in metabolic and toxic maladies is unavailable as of yet. A number of factors such as the rising rates of tourism in the Dead Sea resorts leading to a greater population in danger of poisoning, alongside mounting delinquency with substance abuse and psychopathology with associated suicide attempts each contribute their part [23].

As we have stressed, while the number of procedures per patient remains roughly constant, the gross number of unstable cases dramatically decreased. Currently, we have not witnessed declined function or coping skills with these cases. However, if the observed trend continues we can predict that in the future the need will arise for alternative means for staff to maintain their expertise upon confrontation with urgent cases. We must be sensitive to the possibility that in time students and newly graduated physicians may become less trained in the application of codes and urgent drills. More worrisome is the fact that the proficiency in dexterous skills may gradually decline. Endotracheal intubation, central line catheter insertion such as subclavean or jugular venous access and intravenous pacemaker insertion all demand ongoing experience. To date, the most promising techniques are computer simulations, mannequins and models with options to perform hands-on procedures [24, 25]. These devices should be made available for senior staff to plan regularly scheduled exercise sessions to ensure that teams retain top expertise [26].

### Limitations

We acknowledge a number of limitations to this study. Are we certain that admissions criteria weren’t altered across the examined years? The triage decision to determine whether subjects deserve admission to the shock room raises the question of possible inter-rater variability and personal discrepancies. However, in practically all cases the cut-off distinction is clear as dictated by the ATS (above). The procedure rate in correlation with condition severity per patient was unchanged. The evidence that the general survival and discharge figures were similar during both compared periods is compelling. Nevertheless, there is still a potential for case selection bias and therefore in future studies attention should be paid to eliminate this liability. Finally, overall responsibility across all teams compounded by progressive fear of charges of negligence with firm risk management precautions dictate that under no circumstances would an unstable patient remain in the open triage section of the emergency room under inferior surveillance. Unfortunately the actual files of the patients examined were not available for scrutiny at this time. Had this been possible we may have been able to calculate precise shock indices and evaluate severity upon presentation, a goal we hope to achieve in future prospective trials.

In order to determine whether these results are limited to our unique population or not, they should be compared with those of patients in different geographical locations. If further research supports the finding that the ratio of unstable patients arriving to hospitals is a reliable reflection of population health, it may serve as an aggregate health measure.

## Conclusion

This preliminary study examines an unexplored aspect of emergency care. As a pilot trial the diminishing incidence of unstable patients in the population should serve as an eye opener and encourage further research to verify whether this trend is uniform across the country and perhaps found in other parts of the globe. If similar findings are discovered we should continue to delve into the reasons leading to these welcomed outcomes. Conversely; if this is solely a local phenomenon, perhaps our medical care system can set an example for other medical frameworks.

## Authors’ information

Sody A Naimer has an MD degree from the Ben Gurion University of Negev. He specialized in family medicine and is a director of the family health clinic in Eilon Moreh, Israel, with an additional part-time position as a pediatrician in Kedumim, Israel. He also works part time as a senior emergency medicine physician at the Emergency Medicine Department of the Beilinson MedicalCenter, Petach Tikva, Israel.

Moshe Y Prero has recently completed his MD degree from The Medical School for International Health, Faculty of Health Sciences, Ben-Gurion University of the Negev, Beer-Sheva, Israel.

Tamar Freud has a PhD degree from the Department of Family Medicine of the Ben Gurion University of the Negev. She works as the Research Manager of Siaal Research Center for Family Medicine and Primary Care, Ben-Gurion University of the Negev, Beer-Sheva, Israel.

Bartal Carmi has a MD and MHA degree from the Ben Gurion University of Negev. He specialized in internal medicine, critical care medicine and emergency medicine. He works as the head of the Emergency Medicine Department at Soroka University MedicalCenter, Beer-Sheva, Israel.
